# Psychodynamic Experience Enhances Recognition of Hidden Childhood Trauma

**DOI:** 10.1371/journal.pone.0018470

**Published:** 2011-04-07

**Authors:** David Cohen, Daniel Milman, Valérie Venturyera, Bruno Falissard

**Affiliations:** 1 Service de Psychiatrie de l'Enfant et de l'Adolescent, Hôpital Pitié-Salpêtrière, Paris, France; 2 Centre National pour la Recherche Scientifique, Unité Mixte de Recherche 7222, Université Paris VI, Paris, France; 3 Universités Paris V et XI, Institut National de la Santé Et de la Recherche Médicale, Unité 699, Hôpital Paul Brousse, Paris, France; Royal Holloway, University of London, United Kingdom

## Abstract

**Background:**

Experimental psychology has only recently provided supporting evidence for Freud's and Janet's description of unconscious phenomena. Here, we aimed to assess whether specific abilities, such as personal psychodynamic experience, enhance the ability to recognize unconscious phenomena in peers – in other words, to better detect implicit knowledge related to individual self-experience.

**Methodology and Principal Findings:**

First, we collected 14 videos from seven healthy adults who had experienced a sibling's cancer during childhood and seven matched controls. Subjects and controls were asked to give a 5-minute spontaneous free-associating speech following specific instructions created in order to activate a buffer zone between fantasy and reality. Then, 18 raters (three psychoanalysts, six medical students, three oncologists, three cognitive behavioral therapists and three individuals with the same experience of trauma) were randomly shown the videos and asked to blindly classify them according to whether the speaker had a sibling with cancer using a Likert scale. Using a permutation test, we found a significant association between group and recognition score (ANOVA: p = .0006). Psychoanalysts were able to recognize, above chance levels, healthy adults who had experienced sibling cancer during childhood without explicit knowledge of this history (Power = 88%; p = .002). In contrast, medical students, oncologists, cognitive behavioral therapists and individuals who had the same history of a sibling's cancer were unable to do so.

**Conclusion:**

This experiment supports the view that implicit recognition of a subject's history depends on the rater's specific abilities. In the case of subjects who did have a sibling with cancer during childhood, psychoanalysts appear better able to recognize this particular history.

## Introduction

The concept of the Unconscious was already explicit in philosophy, psychology, neurology and psychiatry long before Freud's and Janet's description of unconscious phenomena [1]. For example, Descartes in “*Les passions de l'âme*” described how unexpected and incomprehensible passions could be related to events during childhood. He stated: “the event is forgotten, the aversion remains”[2]. Although Freud's and Janet's description of unconscious phenomena dates back to the early 1900s [3], [4], experimental psychology has only recently provided supporting evidence of various kinds [5], [6]. (a) Studies of blindsight patients [7] and studies using subliminal masking in normal individuals [8] have shown that non-symbolic stimuli such as emotional faces can induce a modulation of amygdala activity in the absence of conscious perception. (b) Similarly, unconscious semantic processing has been shown in experiments on priming with number words [9], [10] and emotional words [11]. (c) Suppression of conscious unwanted memories by executive control occurred in an experimental set of modified go/no-go tasks [12] and could be correlated to specific neural networks, thus providing a viable model of repression [13]. However, in these experiments, the authors only manipulated conscious suppression and could not validate Freud's concept of unconscious repression that is his genuine original concept of repression. Freud's concept of repression refers to the defensive inhibition of “unbearable” mental content. For him, exclusion from consciousness is effected not simply through suppression (the voluntary form of repression) [14], but also by a variety of distorting techniques, some of which are deployed to degrade latent content, and all of which are eventually subsumed under the rubric of defense mechanisms (the widest sense of repression) [15]. (d) Perceptual learning can occur as a result of exposure to subliminal stimuli without the subject's having to pay attention [16], and this processing may not be passive [17]. (e) Decision-making strategies may involve both conscious and unconscious processing, as demonstrated for complex situations in studies of patients with prefrontal damage [18], and in studies of consumer choice [19]. However, whether specific abilities, such as personal psychodynamic experience, enhance the ability to recognize unconscious phenomena in peers – in other words, to blindly detect knowledge related to individual self-experience – remains one of Freud's most debated postulates.

In the current experiment, we took advantage of several characteristics of the study of childhood trauma. First, according to Freud, individuals usually overcome a traumatic experience through repression [20], [21]. Second, traumatic experiences may be followed by incomplete amnesia or repression that may lead to a spectrum of manifestations including Post-Traumatic Stress Disorder [22]. The syndrome includes both non-specific symptoms (e.g., anxiety, depressed mood) and specific ones such as nightmares and unwanted flashbacks of the traumatic experience that are related to incomplete amnesia. Third, the pattern of amnesia may differ according to cues related to the traumatic experience itself. For example, children abused by a trusted caregiver are more likely to eventually forget the abuse than those maltreated by strangers [23]. Fourth, while mourning for a deceased sibling, clinical experience shows that the survivor may exhibit guilt related to an unconscious wish to evict a competitor [24]. The experimental design also took into consideration the specificity of the analytical situation, which is based on “free association” and usually focuses on intermediate states of consciousness, dreams, and unexpected and unwanted events (e.g., lapses of memory) [25].

We hypothesized that (i) cancer would have a lasting traumatic effect on the siblings of children affected with the disease as it constitutes the accomplishment of an unconscious wish to evict a competitor; (ii) psychoanalysts (PSYANs) should recognize this lasting traumatic effect without explicit knowledge when analyzing free associations collected within a specific experimental paradigm. This last hypothesis was based on the following rationale: first, the work of Freud suggests that basing the interpretation of dreams on free associations is the best way to access an individual's unconscious [3]; and second, psychoanalysts are trained to use free associations and to pay attention to their patients' free associations [25]. For the current experiment, we selected experienced psychoanalysts to maximize the effects of focusing on free associations (see below).

## Methods

### Subjects

Research was approved by the Pitié-Salpétrière ethical committee. The subjects were recruited by advertisement; all were volunteers and gave written informed consent. Seven had a history of a sibling treated for cancer during childhood or adolescence who may or may not have died. Controls were matched for age, gender, socio-economic status and education. An interview determined that controls had not experienced any major traumatic events or loss of relatives. All subjects and controls were screened for current medical and psychiatric diagnoses during a clinical interview. All participants were healthy and had at least two years of university education. The subjects' characteristics are presented in Table 1. There were no significant differences in the socio-demographic characteristics of subjects and controls.

**Table 1:** Characteristics of the subjects (S_i_) and the controls (C_i_).

	Age	Sex	Time^1^	Died^2^	Therapy^3^		Age	Sex	Therapy^3^
**S1**	19	F	10	Yes	No	**C1**	30	F	No
**S2**	35	M	17	Yes	No	**C2**	38	M	Yes
**S3**	25	F	6	Yes	No	**C3**	35	F	No
**S4**	22	M	4	No	No	**C4**	30	M	No
**S5**	24	M	8	No	Yes	**C5**	30	M	No
**S6**	24	F	6	No	No	**C6**	32	F	No
**S7**	29	F	16	Yes	Yes	**C7**	40	F	Yes

1 Indicates the years since the sibling's diagnosis of cancer.

2 Indicates whether the sibling died of the cancer or not.

3 Indicates whether the subjects or controls had had psychotherapy. In the case of S5 and S7, both received psychotherapy because of their trauma. F =  female. M =  male.

There was no significant difference between subjects and controls for age, sex, socio-economic status and education.

### Procedure

All the individuals recruited were asked to give a 5-minute spontaneous speech while being videotaped, following specific instructions (in French). Where applicable, they were also asked not to talk about their sibling's cancer. The instructions were as follows (as translated into English): “*Take roughly 5 minutes to try to talk about how you experience your inner world and your outer world. For instance, you could talk about the importance you assign to your dreams but also about how you relate to art, painting, music, or sculpture, and about the space you give all these feelings in your everyday life. You could also talk about experiences that felt awkward or unexpected to you, such as having the impression of recognizing a place or person you didn't know; or, on the contrary, the feeling of not recognizing a familiar place or person. You could also discuss the nature of your relationship with yourself: are you interested in your inner life, or are you more attracted by external reality? Finally, and this is the most important thing, try to speak freely, saying whatever comes to mind and following your thoughts freely*.”

Three PSYANs were recruited from liaison psychiatry or medical psychology teams. PSYANs were raters who have both received and practiced psychodynamic therapy themselves. To assess whether PSYANs' ability to recognize was specific, we also recruited 4 other sets of three raters: (1) inexperienced professionals (INXPs) were medical students taking elective courses in a university and hospital department of child and adolescent psychiatry; to be sure that motivation and interest in the topic were similar INXPs were selected among student volunteers who planned to specialize in Psychiatry; (2) a second set of INXPs (medical students) was recruited to assess possible framing effect [26] (see below); (3) experienced professionals (EPs) were experienced physicians involved in cancer treatment; (4) cognitive behavioral therapists (CBTs) were recruited from the French association of cognitive behavior therapy. Finally, to assess whether individuals with the same history of sibling's cancer during childhood or adolescence might recognize above chance level individuals with the same trauma by internal echo, we recruited three adults with the same experience (SE) and a post-baccalaureate degree in education (but who did not participate in the video recording and had no professional mental health experience), to participate in the experiment.

All raters were informed of (1) the general principles of the experiment (classification of fourteen videos coming from two groups of 7 subjects; at least 2 hours of availability); (2) that they were expected to do their best in classifying the videos. After acceptance to participate in the study, the subjects were randomly shown each video and asked to classify each one according to whether he/she thought that the speaker had or had not experienced the childhood cancer of a sibling, using a 4-point Likert scale (yes, probably yes, probably no, no). Each rater received the following instructions regarding the experiment and the background hypothesis. The instructions to raters (in French) were created without any knowledge of the contents of the experimental videotapes: “*The question that has been asked is based on the clinical and theoretical hypothesis that cancer would have a lasting traumatic effect on the siblings of children affected with the disease as it constitutes the accomplishment of an unconscious wish to evict a competitor. The instruction given to participants in the videos has been created in order to activate a buffer zone between fantasy and reality. Based on the free association method, it encourages patients to speak, as freely as possible in the circumstances, of their dreams and the importance they attribute to them but also their relationship with art and strange feelings such as* déjà vu. *In order to differentiate between siblings of children with cancer and control subjects, you could search for evidence of the two symptoms we think are most frequent in case of the traumatic realization of the unconscious wish to evict a competitor: (1) Overflowing: in this case, you might look for evidence of confusion, the need to confess, emotional overflow, but also the discreet but pervasive presence of memories or memory fragments that have nothing to do with the question asked in the first place; (2) Withdrawal: we might note a discreet form of extinction here, the silent gaps, and evidence of misunderstanding and repressed sadness. However, and this is the most important thing, you should trust your instinct as a clinician as well as your own assessment criteria. This is particularly important since siblings of children with cancer are notably discreet. During the video, you can go back whenever necessary and watch an earlier scene again. You can change your earlier answers as well*.” Because the language used in the instructions might be more understandable to analytically trained individuals and therefore might have influenced rater responses and scores, we included a second set of INXPs to assess the possible “framing effect” of the instructions given to the raters. This second set of INXPs (the INXP-frame) was simply asked to classify the videos according to the likelihood that the speaker had a sibling with cancer without any additional specific instructions. Similarly, a set of siblings of cancer patients who participated in the experiment were also given the same simplified instructions [26].

### Statistical analysis

To statistically test whether professionals classified cases and controls better than could be expected by chance, we used a permutation test based on a modified version of Fisher's *Lady tasting tea* procedure [27]. This statistical procedure was chosen to limit type I error. The number of cases, controls and raters required to detect differences with power superior to 80% for a p<.05 was calculated. For a sensitivity and a specificity in correctly categorizing each subject, both equal to 80%, 7 cases, 7 controls and 3 raters per group were enough to detect significant differences using the procedure described below with type one error of .05 and power calculated at 88% [28]. It is noticeable that since the raters know that half of the records belong to “cases” and the other half to “controls”, the ratings cannot be considered as independent realizations of a random variable, such that a traditional Student t test or Mann-Whitney test should not be used. On the contrary, under the null hypothesis, cases' and controls' records are indistinguishable; all permutations of scores obtained for each record are equiprobable. Hence, a soundable (one-sided) p.value can be estimated as the proportion of permutations of the n records for which the total score is higher or equal to the total score obtained in the experiment [29]. We used a two-sided p.value based on a similar principle here. Of note, because of multiple testing (6 totally separate p-values were computed empirically), the level for significance was p<0.009. The p-value of the ANOVA combining all groups of raters was estimated from a comparable procedure: the total score is replaced here by the F value of the “group” factor of a two-way ANOVA (subject and group).

Therefore, the association between judge ratings and the actual distribution of subjects into cases and controls was tested in the following way. First, a score was computed for each group of raters: PSYANs, INXPs, EPs, CBTs, SEs, and INXP-frames. This score was obtained by summing all 3*14 coded evaluations: +2 when the raters correctly answered yes or no, +1 when they correctly answered probably yes or probably no, −1 when they incorrectly answered probably yes or probably no, and −2 when they incorrectly answered yes or no. Thus, for each rater, the score could vary from +28 for all correct guesses to −28 for none correct. And, for each group of raters, the score could range from +84 for all perfect to −84 for maximum failure. At this first set, this score is computed for the data set obtained in the experiment. Table 2 shows the age, gender,number of years of experience for health professionals (defined as the number of years since receiving their diploma) and the number of years spent practicing psychotherapy for psychotherapists (defined as the number of years since he or she first supervised patients). Table 2 also shows the score according to each individual subject on video and according to group membership (healthy adults who experienced sibling cancer vs controls).

**Table 2:** Raters' characteristics and scores* according to what extent they recognized or not whether healthy adults (N = 14) experienced sibling cancer during childhood (Subjects) or not (Controls).

		PSYAN			INXP			EP			CBT			SE			INXP-frame		
Rater number		*1*	*2*	*3*	*1*	*2*	*3*	*1*	*2*	*3*	*1*	*2*	*3*	*1*	*2*	*3*	*1*	*2*	*3*
Rater age		48	47	50	25	26	25	41	39	52	35	61	47	26	25	30	24	26	25
Rater gender		M	F	F	F	M	F	F	M	M	F	M	M	F	F	F	F	M	M
Rater years of experience		18	21	19	NA	NA	NA	13	12	24	12	30	21	NA	NA	NA	NA	NA	NA
Rater years of psychotherapy		14	14	10	NA	NA	NA	NA	NA	NA	3	25	17	NA	NA	NA	NA	NA	NA
Subjects	S_1_	+2	+1	+1	−2	−1	−2	+1	+1	+1	−2	−2	−1	+2	+1	+1	+1	+2	−2
	S_2_	+1	+2	+1	−2	+1	+1	+1	−1	+2	−1	+2	−1	+2	+2	+2	+2	+2	−1
	S_3_	+2	+2	+1	+2	−1	−2	−2	−2	+2	−1	+2	−1	+1	+2	−2	+1	−2	+2
	S_4_	+1	+2	+2	−1	+1	−1	+1	+1	+2	+2	−2	+1	+2	−1	−2	−1	+2	+2
	S_5_	+2	+2	+2	+2	+2	+2	+2	+2	−1	+2	+2	+1	+2	+2	+1	+2	+2	+2
	S_6_	+2	+2	+2	+2	−1	+2	+2	+2	+2	+2	−2	+1	+1	+1	+2	+2	+2	−1
	S_7_ **	−1	−1	−1	−1	−1	−2	−2	−1	−1	−1	−1	−1	+1	−2	−1	−2	−2	+1
Controls	C_1_	+2	+2	−2	+2	+2	+2	+2	+1	+1	−2	−1	−1	+1	+1	−1	+2	+2	−2
	C_2_	−1	+1	+1	+1	−1	−1	+2	+2	+2	−2	+2	−1	+1	+1	−2	+1	+2	+2
	C_3_	+2	+1	+1	−2	+2	+2	−2	−2	−1	−2	+2	−1	−2	−1	+1	−2	−2	−2
	C_4_	+2	+1	+1	+2	−1	+1	−1	−1	−2	+1	+2	+1	+1	−2	+1	+1	+2	+2
	C_5_	+2	+1	+1	−2	+2	+2	+2	−1	+1	−2	+1	+1	−2	+1	+2	+2	−2	−2
	C_6_	+2	−1	+1	−2	−1	−2	+2	+2	+2	+2	−2	+1	+1	+2	+2	+1	+2	+2
	C_7_	+1	+2	+1	+2	+2	−2	+2	−1	−1	+1	+1	−1	+1	+1	+2	−2	+2	+2
Total		+19	+17	+12	+1	+5	0	+10	+2	+9	−3	+4	−2	+12	+8	+6	+8	+12	+5

PSYAN: Psychoanalysts; INXP: Inexperienced professional; INXP-frame: Inexperienced professional in the framing effect condition (see method); EP: experienced professional; CBT: cognitive behavioral therapists; SE: same experience; S_i_: Subject 1, 2…or 7 with a history of sibling cancer during childhood; C_i_: Control 1, 2…or 7; NA: Not Appropriate.

*Scores are obtained as follows: +2 when the rater correctly answered yes or no according to whether or not each healthy adult experienced sibling cancer during childhood, +1 when they correctly answered probably yes or probably no, −1 when they incorrectly answered probably yes or probably no, and −2 when they incorrectly answered yes or no.

**Notably, it is the same case subject who was incorrectly classified into the control group by all three POPs.

**Figure 1:**
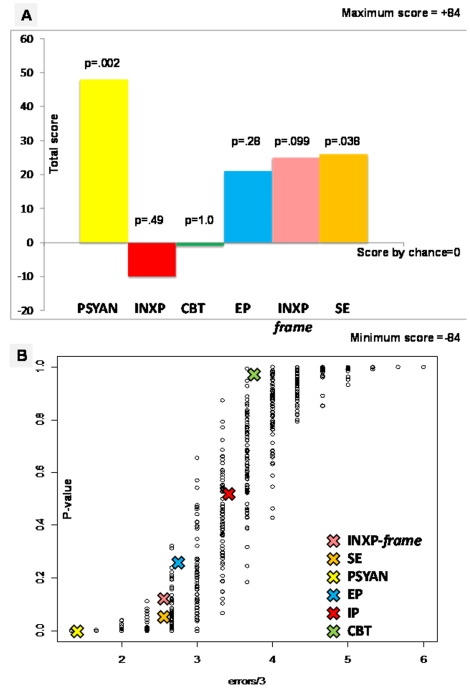
Main results. **A: Recognition scores of each rating group.** Psychoanalysts [PSYAN], inexperienced professionals' [INXP] in similar and simple rating instruction condition [so called INXP-*frame*], cognitive behavioral therapists [CBT], experienced professionals [EP], and individuals who had the same experience of history of sibling's cancer [SE] scores when determining whether healthy adults had experienced sibling cancer during childhood, without explicit knowledge of this history. For each group, the score could vary from +84 for all perfect guesses to −84 for a complete failure and the probability that the score differed from chance was calculated using a permutation test. ANOVA combining all groups of raters: p = .0006. Computed p-value for each group of raters is indicated upon the bar (level of significance p<.009). **B: Recognition p-values as a function of the mean number of errors per judge.** To give an idea of the variability for each level of performance in terms of group recognition, we performed a simulation with judges having random errors and calculated the possible p values. The curve gives an idea of the p value as a function of the mean number of errors per judge, whereas the plot dispersion (vertical) reflects the variability of the p value given all possible changes in unknown parameters. Each experimental result is indicated with a large cross and superimposed on the plots curve using the same acronyms as those in figure 1A.

To find out whether a particular group classified cases and controls better than could have been expected by chance, a permutation test was done as described above using R software version 2.4.1 [30]. The p-value was finally equal to twice the number of permutations for which the scores were above the score obtained for the original data set in the experiment. Given that we used a modified version of the Lady Tea Test procedure, it is not possible to provide a table showing the number of permutations for each level of performance on the dyads, because first of all there are three judges and secondly, possible answers are not yes or no but +2, +1, −1 and −2 (see table 2). Therefore, the number of possible errors ranges between 0 and 42 (2*21). To give an idea of the variability for each level of performance, we performed a simulation with judges having random errors and calculated the p value (see figure 1B).

## Results

The results are summarized in figure 1. Using an ANOVA combining all groups of raters, we found a significant association between group and total score (p = .0006). All three PSYANs were able to correctly classify 12 out of 14 subjects and controls, which is far above the chance level (Power = 88%; p = .002) with a specificity and a sensitivity equal to 0.86. In contrast, all other groups of raters (INXPs, EPs, CBTs) were unable to do so. To assess possible framing effect [26], three other INXPs who received simplified instructions performed the experiment and did not differentiate from chance. To assess possible internal echo, three individuals who had the same history of a sibling's cancer, so called same experience (SE) also performed the same experiment with simplified instructions and did not differentiate from chance.

Given these intriguing results, a qualitative analysis of the responses was conducted. (a) Interestingly, it was always the same subject (S7 table 2) with a history of sibling loss that was incorrectly classified by all PSYANs, whereas the controls incorrectly identified differed for each PSYAN. (b) To investigate whether experience with psychotherapy may partially explain the results, we specifically asked the subjects participating in the videos about any previous experience they might have had. Four subjects had received psychotherapy during childhood or adolescence for several months, two in each group (Table 1). (c) Only one subject reported a subjective improvement due to treatment. This was the person who was incorrectly classified by all three PSYANs. These data, together with the fact that CBT did not differentiate from chance, do not support the hypothesis that PSYANs recognized adults with a history of sibling cancer better because of their more extensive experience with psychotherapy. However, given that it might be argued that the cognitive style developed during psychotherapy may differ according to the nature of the problem to be treated, one may imagine that recording from these four subjects might bias such cues. To assess this possibility, we performed a secondary analysis including only the 10 subjects without experience of psychotherapy. PSYANs were still able to recognize, above chance levels, healthy adults who had experienced sibling cancer without explicit knowledge of this history (p = 0.008).

## Discussion

### General comments

In very recent years, there has been renewed interest in psychoanalytic theory thanks to the neuroscience of awareness [1], [31]. Most of the experimental data are related to basic cognitive processing, with the notable exception of the psychology of decision-making [19], [26] and of the psychology of social influences [32]. In these studies, the experimental tasks used paradigms from cognitive psychology and manipulated variables in order to be sure that parts of the raters' responses were secondary to unconscious stimuli. Such manipulations could have focused on the duration of stimuli presentation, stimuli masking and/or the use of unconscious primes [15]. The possible consequences of unconscious processing were evidenced by different reaction times during experimental tasks and/or by brain functional imaging [5]–[13]. Other studies were based on a neuropsychological paradigm where patients had specific consequences of brain damage [18]. Consequently, although these studies support Janet's and Freud's discovery of unconscious processing, they do not support other aspects of Freud's psychoanalytic theory. For example, is the brain network that has been reported to play a role in suppression [12], [13] also implicated in psychodynamic repression? Are the neural mechanisms underlying visual perceptual suppression related to those underlying psychodynamic repression [14]? Are Freud's descriptions of memory distortions due to repression essentially the same as those reported in Barlett's research on biographical memory, which was shown to be reconstructive and elaborative? For Erdelyi, Barlettian and Freudian reconstructions are essentially the same, even in name, differing only in nature (cognitive vs. emotional) [15]. Although Rao and Keshavan reported evidence to support better recognition of bipolar disorder by psychiatrists in paintings [33], to the best of our knowledge, we report here on the first experimental evidence using a psychoanalytic paradigm showing that a specific quality of a rater (here being psychoanalysts) enhances the ability to process unconscious phenomena in peers – in other words, to detect, far above chance levels, subject's status related to traumatic individual experience without explicit knowledge of this history. Our experiment supports the view that intuitive recognition of a subject's history depends on the rater's specific abilities. In the case of trauma, PSYANs appear to be better able to recognize this particular history. This ability appeared to be rather specific as we had several rater control conditions.

The experimental paradigm was based on the psychoanalytic theory of trauma as illustrated in the procedure. First, we selected a traumatic event involving a sibling to ensure both its traumatic impact and its specific internal echo regarding the unconscious wish to evict a competitor. Second, we selected adults who have likely experienced the effects of repression from the time of the traumatic event to the time of the current experiment. Third, to facilitate accessing unconscious traces, participants who gave the 5-minute spontaneous free-associating speech received specific instructions designed to activate a buffer zone between fantasy and reality. Fourth, raters (except INXP-frame) received instructions on the experiment and the background hypothesis. However, how the PSYANs actually formulated their choices needs to be explored and the experiment as such cannot demonstrate the psychoanalytic theory of trauma. Taking into account recent developments in (i) unconscious and conscious thought theory, on one hand, and (ii) mathematical models of psychic processing, on the other, some speculations can be made.

### Is it possible to model unconscious interpersonal exchanges?

The unconscious and conscious thought theory [34] aims to explain the psychological processes humans associate with thought, such as decision-making, choosing, impression formation and creativity. The theory maintains that unconscious and conscious thoughts have different characteristics, making them differentially applicable in a variety of situations. There are arguments to support the hypothesis that unconscious thought is not passive but active, and is more creative (producing more original items), associative and divergent than conscious thought [35]. Regarding the quality of unconscious thoughts, alternative hypotheses have been proposed. Instead of the superiority of unconscious information processing, some authors have reported data indicating that excessive deliberation can deteriorate high-quality first impressions [36]. Mathematical models of psychic processing constitute another attempt to model the complexity of human thought [37]. At a very basic level, neural networks are best modeled as networks of binary automata that are on or off over a course of time. Using Hebb's rule, memorization processing is not modeled as memory sticks or boxes, as in computer science, but rather as network configurations that are stabilized through development thanks to probabilistic learning. The more frequently an object occurs, the more the corresponding network configuration is activated, leading to neural commitment and a “magnet effect.” These proposals have been supported by artificial intelligence studies that were able to simulate face recognition [38], and by psycholinguistic studies that demonstrated neural commitment through probabilistic learning of phonetic units of language [39]. Studies of babies' attention to sounds that compared languages and computerized subtle modifications of phonemes provided an experimental illustration of the magnet effect as a result of probabilistic learning [39]. To handle the complexity of human thought, the mathematical model of thought proposes a meta-organization of several networks including sensory input, sensory integration, sensory association, consciousness, action, intentionality, language, emotionality, etc., that are interconnected and somehow hierarchical [37]. One of these networks is related to consciousness and acts as a workspace framework that confers a feeling of being conscious when activated [40]. Given that direct interconnections between pairs of networks exist, automatic actions or thoughts without awareness are allowed.

In the current experiment, we may speculate that PSYANs, given their personal experience with psychoanalysis, were more able to make choices dependent on more associative and divergent thought, allowing them to intuitively recognize individuals' self-experience at above chance levels. However, there was no time pressure on choice making during the experiment and PSYANs were also involved in conscious processing to formulate choices [19], [36]. As an alternative hypothesis, we can speculate that the PSYANs recognized childhood trauma better because of subtle consequences such as the specific use of particular defense mechanisms, as indicated in the instructions. In other words, if we link the two preceding hypotheses, we can speculate that PSYANs activated an internal echo of traumatic experiences – developed during their experience with psychoanalytic therapies – that helped them to formulate a choice.

### Limitations of the study and further research

First, the experimental procedure was designed in order to detect large differences between accurate response and chance, and between groups. Given the relatively small sample size of the current study, we cannot exclude that other groups of raters could also recognize childhood trauma at a subtle level only detectable with larger group of raters. Regarding the question of whether an internal echo helped the decision-making process, we would expect that subjects with a similar experience of sibling cancer during childhood recognized the cases at above chance levels. In the current experiment they only show a tendency to reach significance (p = .038, but the significance due to multiple testing was p≤.009) that may be related to a smaller effect.

Second, given the study protocol, it is not possible to distinguish implicit recognition or knowledge from unconscious communication. Similarly, we can not exclude that the above chance ratings we observed with PSYANs were due to unconscious experimenter bias. Some other sources may be fruitful to explore how this unconscious or implicit information was conveyed in interpersonal exchanges. Comparisons of cases' and controls' speech in terms of speech content, linguistic cues, prosody, rhythm may help distinguishing – if differences are found – what PSYANs had intuitively perceived. These ambiguities could be clarified by future studies in which PSYANs are asked how they had formulated their choices, but this was not performed in the current experiment.

Third, because we only selected psychoanalysts with extensive training and experience, we cannot determine the extent to which training and experience with free associations are important. To test this hypothesis, we aim to examine a large panel of psychoanalysts and determine whether recognition scores correlate with age or training.

Fourth, although the study protocol was based on the psychodynamic theory of trauma, the results do not demonstrate the theory, as we don't know the parameters that are involved in unconscious communication, or the extent to which one needs a special theory of trauma to accurately classify cases and controls. Furthermore, we cannot exclude that even those with an opposing/different theory can detect something but with a smaller effect.

### Conclusion

In sum, this experiment supports the view that recognition of a subject's history depends on the rater's specific abilities. In the case of subjects who had had a sibling with cancer during childhood, PSYANs appear better able to recognize this particular history. How they actually process this knowledge needs to be explored.
